# Protective effect of *Urtica dioica* leaf hydro alcoholic extract against experimentally-induced atherosclerosis in rats

**Published:** 2018

**Authors:** Fatemeh Namazi, Tahoora Shomali, Pouya Taghikhani, Saied Nazifi

**Affiliations:** 1 *Department of Pathobiology, School of Veterinary Medicine, Shiraz University, Shiraz, Iran*; 2 *Division of Pharmacology and Toxicology, Department of Basic Sciences, School of Veterinary Medicine, Shiraz University, Shiraz, Iran*; 3 *Department of Clinical Sciences, School of Veterinary Medicine, Shiraz University, Shiraz, Iran*

**Keywords:** Atherosclerosis, Stinging nettle, Oxidative stress, Histopathology, Urtica dioica, Rat

## Abstract

**Objective:**

Finding compounds that could be used for prevention of atherosclerosis (AS) is highly desired. The present study evaluated the protective effects of *Urtica dioica* (UD, commonly known as stinging nettle) leaf ethanolic extract against high-fat diet-induced AS in rats.

**Materials and Methods:**

In this study, 40 male adult Sprauge-Dawley rats were randomly allocated to 4 equal groups and treated as follows for 9 consecutive weeks: (1) Normal control (NC; normal rats that were fed with a basic diet); (2) Atherosclerotic rats (AT; which received no particular treatment); (3) Atherosclerotic rats that received 100 mg/kg/day ethanolic extract of UD orally and (4) Atherosclerotic rats that received simvastatin 4 mg/kg/day orally. Atherosclerosis was induced by a high-fat diet accompanied by propylthiouracil and vitamin D3.

**Results:**

Marked hypercholesterolemia and significant increase in LDL-C/HDL-C ratio were observed in rats of AT group. Administration of UD significantly reduced these parameters as compared to AT group (p<0.05 for all cases). In histopathological evaluations of the aortic arch, AT rats showed atherosclerotic lesions, which were markedly ameliorated in rats treated with UD or simvastatin. Simvastatin and UD significantly reduced medial (p<0.05) but not intimal thickness. Increased level of malondialdehyde (MDA) and reduced total antioxidant capacity (TAC) were observed in the aortic arch of AT rats (p<0.05 for all cases). In contrast with simvastatin, UD extract had no significant effect on these parameters.

**Conclusion:**

Ethanolic extract of UD prevents establishment of atherosclerotic lesions in rat aorta, which is associated with positive effects on serum lipid profile without significantly affecting antioxidant status.

## Introduction

Atherosclerosis (AS) is associated with thickening and hardening of the arteries' walls, which generally predisposes the patient to severe cardiovascular events such as heart attacks, strokes, and acute coronary syndrome due to disturbances in blood flow when enough narrowing or closure of arterial lumen occurs. Although not completely elucidated, the pathological process underlying AS is initiated by endothelial damage which results in exposure of proteoglycans with binding affinity for Apo B100 moiety of LDL particles. This leads to accumulation of LDL in intima and subsequently causes oxidative states by increased levels of reactive oxygen species or oxidative enzymes released by inflammatory cells. These oxidized lipids give rise to over expression of adhesion molecules and secretion of pro-inflammatory cytokines that promote generation of 'fatty streaks' made of T cells and foam cells loaded with lipids. The process can progress to the evolution of a necrotic core (Khan et al., 1995 ▶; Weber and Noels, 2011 ▶). 

Administration of high-fat diets to promote AS in animal models is a valuable tool for studying pathogenesis and testing novel compounds for treatment or prevention of AS. Rats and mice are not good models for AS, because they are typically resistant to atherogenesis; therefore, other interventions, such as administration of vitamin D_3_, to promote vascular calcification is often required (Leong et al., 2015 ▶) which considerably elevates the rate of lesion generation. 

Unfortunately, currently available therapeutic approaches (e.g. statins as the most appreciated and routinely prescribed therapeutics for AS), do not completely address the inflammatory processes responsible for progression of AS. Established therapeutic agents used for AS mainly focus on alleviating hypertension and hyperlipidemia or controlling homeostasis to prevent thrombotic complications (Weber and Noels, 2011 ▶). Therefore, discovery of multifaceted compounds with high safety and appreciable outcome is still intriguing; especially, compounds that can prevent establishment of atherosclerotic lesions are highly desired. 

Medicinal plants comprise an underexploited source of drugs. *Urtica dioica* L. (UD) (Urticaceae) or “stinging nettle” abundantly grows in northern Europe and much of Asia and is usually found in the countryside. This herb has a history of use in traditional medicine to treat diverse conditions including lipid disturbances. Positive effect of UD against lipid derangements has been demonstrated in *in vivo* studies (Avci et al., 2006 ▶ and Daher et al., 2006 ▶). More recently, a triplex mixture of *Peganum* *harmala*, *Rhuscoriaria*, and UD aqueous extracts was shown to improve metabolic and histological parameters in diabetic rats (Abedi Gaballu et al., 2015 ▶) and in a study done by Nassiri-Asl et al., 2009 ▶, UD extract, especially at 100 mg/kg, showed positive effects on serum lipid profile and liver histopathological features of rats fed with a high-cholesterol diet. This motivated us to evaluate the plausible protective effects of UD against establishment of atherosclerotic lesions in animals fed ahigh-fat-diet as an animal model of AS.

## Materials and Methods

**Preparation of the extract**

Dried leaves of UD were purchased from an authentic local medicinal herb shop (Shiraz, Iran, May 2016) and after confirmation of quality, genus and species by a botanist (Shiraz University, School of agriculture), were used for extraction. The extraction technique was cold maceration by using 70% ethanol as the solvent. Briefly, 100 g of finely ground leaves was macerated in 1000 ml of 70% ethanol (herb/solvent ratio of 1:10 w/v) for 72 hr with intermittent stirring. After filtering, the solvent was evaporated in a rotary evaporator at 50°C. The powder was kept at -20 °C until use. The powder was dissolved in distilled water at the time of administration. The herb/extract ratio was 8:1 (w/w) (The extraction method was previously described by Nassiri-Asl et al., 2009 ▶ and done following minor modifications).

**Animals and study design**

Forty male adult Sprauge-Dawley rats with a mean body weight of 200±15 g were adapted to standard environmental conditions, including 12hr/12hr light/dark cycles at 23±2 °C for one week and then were randomly allocated to 4 equal groups (n=10 in each) and treated every day for 9 consecutive weeks as follows: 

1. Normal control (NC) (normal rats that were given a commercial corn-soy based diet as basic diet with the analysis presented in Table 1).

2. Atherosclerotic rats (AT) (rats that received no particular treatment).

3. Atherosclerotic rats that received 100 mg/kg/day ethanolic extract of UD by oral gavages (UD group). The dosage was chosen based on the results of the study done by Nassiri-Asl et al., 2009 ▶ for hypolipidemic effects of UD hydro alcoholic extract.

4. Atherosclerotic rats that received simvastatin (Shahr Darou Ltd., Iran) 4mg/kg/day by oral gavages (comparative control or CC group) (Hu et al., 2015 ▶).

In this study, AS was induced by administration of high-fat diet along with propylthiouracil (PTU) and vitamin D_3_. The high fat diet contained 80.8 % normal diet, 3.5 % cholesterol (Sigma, USA), 10 % animal oil (sheep ghee), 0.2 % PTU (Iran Hormone Co., Iran), 0.5 % sodium cholate (Sigma, USA), and 5 % refined sugar. Moreover, rats received vitamin D_3_(100,000 IU/kg; Osve pharmaceutical Co., Iran) during the first four days by oral gavages. AS induction was done according to the reports of Hu et al., 2015 ▶ and Pang et al., 2010 ▶, with little modifications.

At the end of the 9^th^ week and after an overnight starvation, all animals were weighed, bled by cardiocentesis under diethyl ether anesthesia and then sacrificed by deep anesthesia. One cm-long samples were immediately removed from the aortic arch of all rats. 

All procedures used in the present study were done in accordance with institutional ethical guidelines for the care and use of laboratory animals in experiments compatible with European convention for the protection of vertebrate animals used for experimental and other scientific purposes.

**Serum lipids assays**

The levels of TC, TG, HDL-C, and LDL-C in serum were assayed using commercial kits prepared by Pars Azmoon Co., Iran as instructed by the manufacturer. Total cholesterol and TG were assayed by cholesterol oxidase-phenol+ aminophenazone (CHOD-PAP) and glycerol-3-phosphate oxidase- phenol+ aminophenazone (GPO-PAP) colorimetric methods, respectively. Enzymatic methods were used for determination of LDL-C and HDL-C concentrations. 

**Histopathological and histomorphometric evaluation of aortic arch**

Half of the aortic arch samples were fixed in 10% neutral buffered formalin. After routine histological processes, 5-μm thick longitudinal sections were prepared and stained with hematoxylin and eosin to be examined under light microscope.Then, photomicrographs were prepared and thickness of tunica intima and tunica media in each sample was determined as the mean of 3 measurements in one microscopic slide by using Zeiss Axio Vision 4.8 software. 

**Determination of the aortic arch malondialdehyde (MDA) content and total antioxidant capacity (TAC)**

The remaining part of the aortic arch was kept in -70°C until use. Samples were mechanically homogenized in 1/1 (w/v) PBS 100mM (pH 7.4) and then sonicated on ice 3 times (each time for 20 sec) with 20-sec intervals, followed by centrifugation at 15000 rpm for 10 min at 4^∘^C. The supernatant was used for determination of TAC and MDA content by colorimetric method at 490 and 535 nm respectively, using Zell Bio kits, Germany. 

**Statistical analysis**

Data were expressed as mean±SD and subjected to one-way analysis of variance (ANOVA) followed by Tukey's multiple comparison test. A p<0.05 was considered statistically significant (SPSS software version11.5).

## Results

**Body weight and serum lipids**

Marked hypercholesterolemia was observed in rats of AT, UD and CC groups that received a high-fat diet as compared to NC group (p<0.001 for all comparisons). Interestingly, simvastatin administration only insignificantly reduced hypercholesterolemia in rats of CC group, while administration of UD significantly diminished hypercholesterolemia in comparison to AT group (p<0.01). 

**Table 1:** Analysis of the composition of the basic diet

**Humidity (%)**	9.9
**Dry matter (%)**	90.1
**Crude fat (%)**	3.4
**Crude protein (%)**	21.5
**Crude fiber (%)**	3.4
**Ash (%)**	5.35
**NaCl (%)**	0.37
**Calcium (%)**	0.93
**Phosphorus (%)**	0.73
**Nitrogen-free extract (%)**	56.4
**Total digestible nutrients (%)**	75.5
**Digestible energy (Kcal/Kg)**	3330
**Metabolisable energy (Kcal/Kg)**	2910

**Table 2:** Serum lipid parameters and body weight (mean±SD) of rats in different groups (n=10).

	**NC**	**AT**	**UD**	**CC**
**Total cholesterol (mg/dl)**	51.7±8.76**	618±139	455±89*	569±119
**LDL-C/HDL-C**	0.421±0.094**	2.02±0.521	1.25±0.270*	1.88±0.506
**Triglycerides (mg/dl)**	64.5±11.6	63.8±17.2	53.2±8.17	58.2±14.7
**Body weight (g)**	290±23.2**	225±24.2	218±15.4	202±13.5

Asterisk signs are used to show significant difference with AT group

NC: Normal control (normal rats that were fed with a commercial corn-soy-based diet); AT: atherosclerotic rats that received no particular treatment; UD: atherosclerotic rats that received 100 mg/kg/day ethanolic extract of UD orally and CC: atherosclerotic rats that received simvastatin 4 mg/kg/day orally, for 9 weeks.

* P<0.01,

** P<0.001

Rats in AT group showed a significant increase in LDL-C/HDL-C ratio with about 5-time increase as compared to NC rats (p<0.001). Administration of UD significantly reduced this parameter as compared to AT group (p<0.01), whereas simvastatin showed a slight reducing effect. Serum TG levels were not statistically different among different groups (p>0.05). Rats in AT, UD and CC groups showed statistically non-significant differences in terms of body weights which was significantly lower than normal animals in NC group (p<0.001), (Table 2). 

**Histopathological evaluations**

No lesion was observed in the aortic sections of NC group, while the rats of AT group showed degeneration of tunica intima, deposition of lipid and formation of the plaques. Tunica media was also thickened.

**Figure 1:**
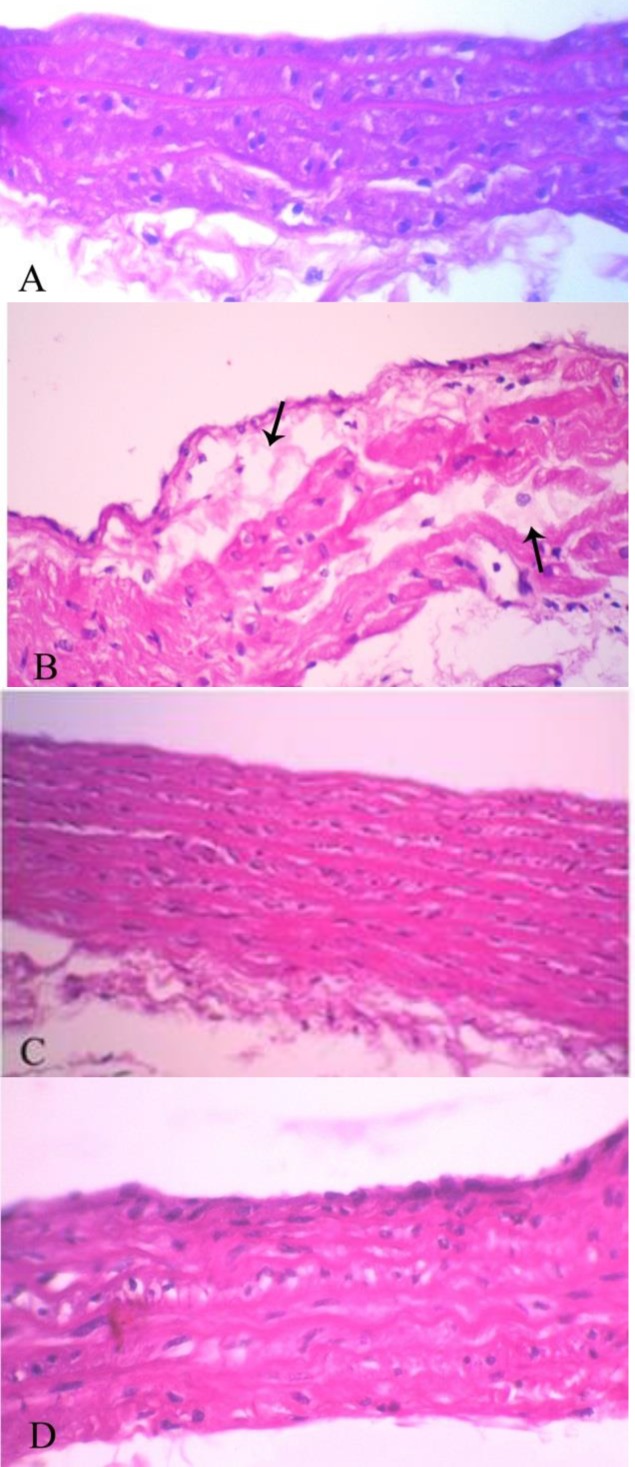
(A) Aortic tissue section of a rat from normal control group shows normal architecture. (B) Tissue section of a rat from atherosclerotic group reveals deposition of lipid (arrows). (C) and (D) show tissue sections of groups treated with simvastatin and stinging nettle, respectively with no damage or lipid deposition (H&E X100

In other groups, tissue sections were normal and no damage or thickening was seen (Figure 1). 

**Thickness of tunica intima and tunica media of the aortic arch**

**Figure 2:**
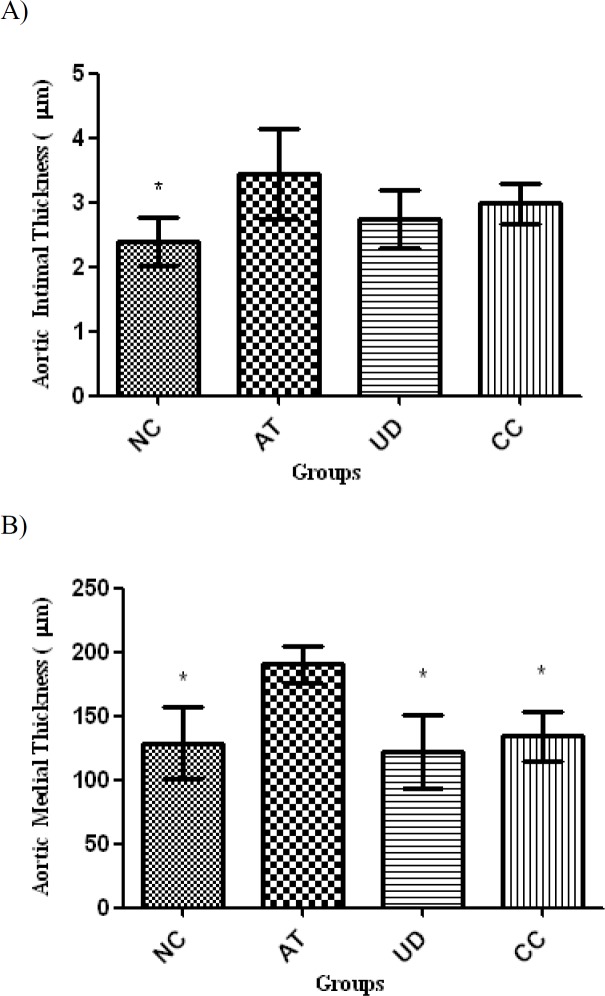
The aortic arch intimal (A) and medial (B) thicknesses (mean±SD) in different groups (n=10). Asterisk sign is used to show significant difference with AT group (*P<0.01). NC: Normal control (normal rats fed with a commercial corn-soy-based diet); AT: atherosclerotic rats that received no particular treatment; UD: atherosclerotic rats that received 100 mg/kg/day ethanolic extract of UD orally and CC: atherosclerotic rats that received simvastatin 4 mg/kg/day orally for 9 weeks

The aortic arch intimal and medial thicknesses of different groups are shown in Figure 2. Both tunica intima and tunica media had higher thicknesses in AT group as compared to NC rats (p<0.05 and p<0.01, respectively). Although UD as well as simvastatin administration decreased intimal thickness, the reduction was not significant in comparison with AT group. In medial layer, however, there was a significant decrease in thickness of rats in groups treated with UD or simvastatin as compared to AT animals (p<0.01 and p<0.05, respectively). Interestingly, rats of these two groups showed statistically comparable medial thickness as compared to NC group.

**Aortic arch MDA content and TAC**

**Figure 3:**
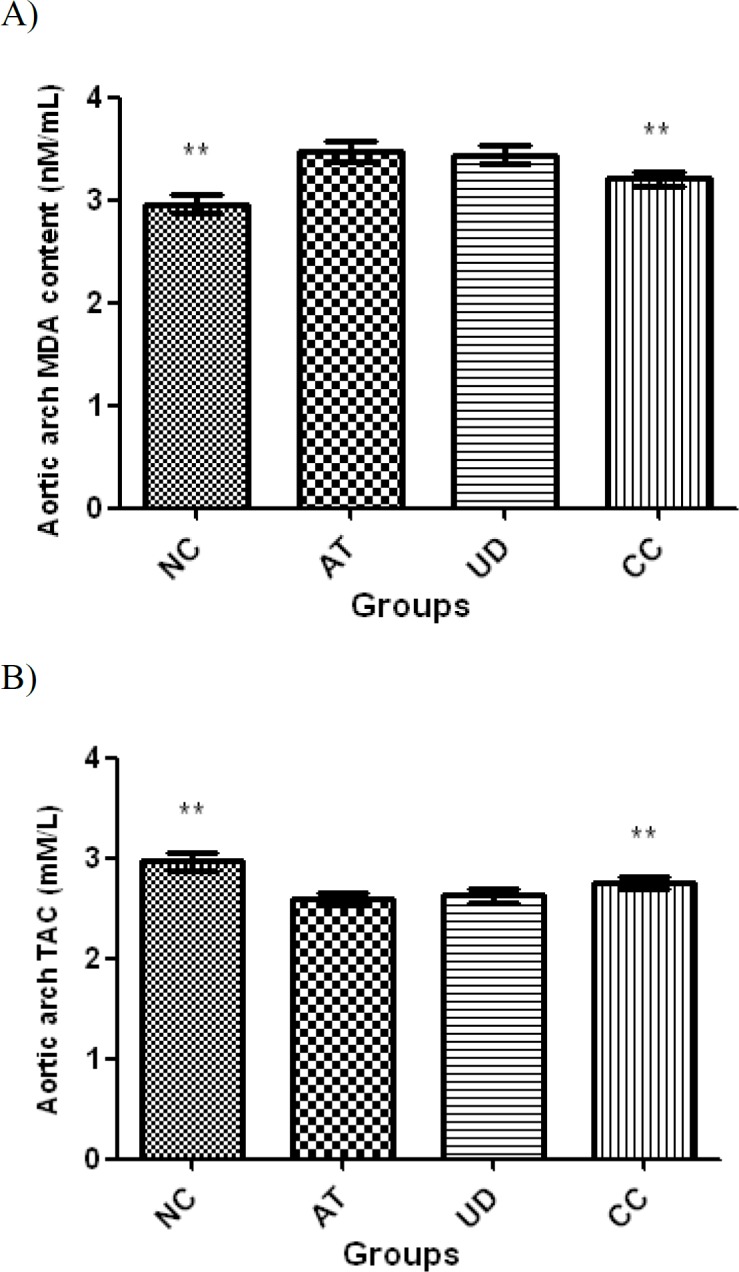
Aortic arch MDA content (A) and TAC (B) (mean±SD) in different groups (n=10). Asterisk sign is used to show significant difference with AT group (**P<0.001). NC: Normal control (normal rats fed with a commercial corn-soy-based diet); AT: atherosclerotic rats that received no particular treatment; UD: atherosclerotic rats that received 100 mg/kg/day ethanolic extract of UD orally and CC: atherosclerotic rats that received simvastatin 4 mg/kg/day orally for 9 weeks

As demonstrated in Figure 3, induction of AS was associated with an oxidative stress in the aortic arch demonstrated by an increased level of MDA accompanied by reduced TAC in rats of AT group as compared to NC rats (p<0.001 for both comparisons). Administration of UD extract had only a slight ameliorative effect on these parameters as compared to AT group (p>0.05). Simvastatin administration in CC group significantly increased TAC and reduced MDA content as compared to AT rats (p<0.001 for both comparisons); however, these parameters were not restored to normal levels of NC group (p<0.001 for both parameters). 

## Discussion

The current study confirms positive effects of the UD hydro alcoholic extract on serum lipid parameters; also, this study, for the first time, indicated UD protective effects against establishment of atherosclerotic lesions in a rat model of dietary-induced AS with regard to histomorphometric and histopathological features and clearly showed that these positive effects are not associated with the antioxidant effect of UD extract in the aortic arch.

Atherosclerosis is among the leading causes of death worldwide; however, rigorous works of scientists in order to find agents that can reduce cardiovascular events due to this condition, are still far from perfect (Gleissner, 2016 ▶). The demand for finding new and efficient drugs is addressed by many researchers in studies focused on herbs with well-known positive effects on serum lipid profile derangements as a classic risk factor for atherogenesis. Stinging nettle has shown to be promising in controlling metabolic diseases which are associated with derangements in lipid profile in both animal models and human studies. Administration of UD has reduced serum TG, TC and LDL-C while increased HDL-C levels in an animal model of polycystic ovary syndrome (Zare et al., 2015 ▶). In a relatively small, single-blind randomized clinical trial, Tarighatesfanjani et al. (2012) ▶ observed that administration of hydro alcoholic extract of UD 100/kg/day to type 2 diabetic patients for 8 weeks, is associated with a significant reduction in TG as well as LDL-C/HDL-C and TC/HDL-C ratios accompanied by an increase in HDL-C levels (Tarighatesfanjani et al., 2012 ▶). In a closely related study, aqueous extract of UD (150 mg/kg/day administered for a month) improved blood lipid profile in rats fed with high-fat diet (Daher et al. 2006 ▶). It should be mentioned that Daher et al. used a model of relatively mild hyperlipidemia by adding 5% w/w coconut oil to rats' diet for only a month. Due to the natural resistance of rats to atherosclerotic lesions, it does not seem that this intervention could induce AS in rats, a matter that has not been addressed by the authors. In the present study, in addition to hyperlipidemia, rats showed overt signs of AS in the aortic arch with regard to histopathological results which was due to the complex regimen used to induce the AS lesions. Consistent with others, we observed an ameliorative effect for UD extract on dyslipidemia (which was surprisingly more pronounced than simvastatin), although we could not determine the underlying mechanism of this effect. Daher et al. observed that administration of UD is not associated with a change in fecal TG or cholesterol content in high-fat-diet fed rats; therefore, prevention of fat absorption does not seem to be responsible for hypolipidemic effect of UD. This promotes the idea that hypolipidemic effect of UD may be due to its plausible effect on lipoprotein metabolism. It is worth to mention that similar to the results reported by Daher et al., UD-induced reduction in LDL-C/HDL-C observed in our study, was basically the result of direct reduction in LDL-C levels where HDL-C levels of rats treated with UD were statistically comparable to those of AT rats. Reduction in apo B synthesis by UD was previously described by Daher et al. and may be responsible for this effect. Other possible mechanisms need to be clarified in future studies. 

In a pioneer study performed by Joris et al. in 1983 ▶ on the pathogenesis of AS in the aorta of hypercholesterolemic rats, the authors reported that in this model, atherosclerotic plaques are initiated by mononuclear cell adhesion and emigration followed by the emergence of sub endothelial foam cells and fatty streaks and typical atherosclerotic plaque formation. Endothelial denudation was not a necessary step in the pathogenesis. High levels of LDL-C decrease nitric oxide production (Feron et al., 1999 ▶). Resultant vascular smooth muscle proliferation and leukocyte adhesion are important in atherogenesis and medial thickening (Kawashima and Yokoyama, 2004 ▶). In the present study, we also observed thickening of tunica intima and media of rats in AT group which was reduced to a statistically similar degree by both UD extract and simvastatin. This decrease was more pronounced for medial thickness. In 2001, Kaneider et al. showed that cerivastatin is able to inhibit leukocyte chemotaxis and has apoptotic effects on neutrophils, monocytes and smooth muscle cells of the vascular wall. This can describe the reducing effect of simvastatin on intimal and medial thickness of atherosclerotic rats in our study; nonetheless, whether the same mechanism is applicable for UD remains to be clarified in future studies. 

Production of reactive oxygen species (ROS) by endothelial, vascular smooth muscle, and adventitial cells is enhanced in AS and these ROS in turn, can promote atherogenesis especially by oxidative modification of lipoproteins and phospholipids (Li et al., 2014 ▶). As stated in the present study, the increase in MDA accompanied by decreased TAC in the aortic arch of rats in AT group, indicate the occurrence of oxidative stress in this group. In contrast to simvastatin, UD extract did not ameliorate the severity of oxidative stress. Antioxidant properties of statins have been well established. In addition to their lipid-lowering ability, statins can reduce production of ROS and increase the resistance of LDL to oxidation. These properties enhance their preventive effect in atherosclerotic disease (Rosenson, 2004 ▶). Both oxidant and antioxidant properties of UD have been reported. In a previous study ROS formation and lipid peroxidation were concentration-dependently increased following treatment of human gastric (MKN45) and colon (HT29) cancer, as well as normal human foreskin fibroblast (HFF) cells with UD hydro alcoholic extract (Ghasemi et al., 2016 ▶). On the contrary, in a clinical trial on patients with type 2 diabetes, Namazi et al., 2012 observed that after eight-week administration of hydro alcoholic extract of UD, blood levels of TAC and superoxide dismutase (SOD) activity increased without a change in MDA and glutathione peroxidase (GPX) activity compared to base line (Namazi et al. 2012 ▶). 

One of the limitations of our study is that we did not perform phytochemical evaluation of the extract; however, in a previous study performed by Mojab et al., 2003 ▶ it was shown that hydro-alcoholic extract of UD contains alkaloids and saponins while it is void of flavonoids and tannins which are famous for their antioxidant properties (Mojab et al., 2003 ▶). In another study, it has been shown that the ethyl acetate fraction of UD has potent antioxidant activities as compared to other fractions (Bisht et al., 2016 ▶). Therefore, the method of extraction or the tissue or site assayed may have affected the antioxidant properties of UD. 

In conclusion, our study showed that hydro alcoholic extract of UD prevents establishment of atherosclerotic lesions in rat aorta, which is associated with positive effects on serum lipid profile without significantly affecting antioxidant status. 
